# Reducing LGBTQ+ adolescent mental health inequalities: a realist review of school-based interventions

**DOI:** 10.1080/09638237.2023.2245894

**Published:** 2023-08-17

**Authors:** Elizabeth McDermott, Alex Kaley, Eileen Kaner, Mark Limmer, Ruth McGovern, Felix McNulty, Rosie Nelson, Emma Geijer-Simpson, Liam Spencer

**Affiliations:** a School of Social Policy, University of Birmingham, Birmingham, UK; b School of Health and Social Care, University of Essex, Colchester, UK; c Population Health Sciences Institute, Newcastle University, Newcastle upon Tyne, UK; d Faculty of Health and Medicine, Lancaster University, Lancaster, UK; e School of Sociology, Politics, and International Studies, University of Bristol, Bristol, UK

**Keywords:** LGBTQ+, youth, adolescent, schools, mental health

## Abstract

**Background:**

LGBTQ+ young people have elevated rates of poor mental health in comparison to their cisgender heterosexual peers. School environment is a key risk factor and consistently associated with negative mental health outcomes for LGBTQ+ adolescents.

**Aims:**

To examine how, why, for whom and in what context school-based interventions prevent or reduce mental health problems in LGBTQ+ adolescents.

**Methods:**

A realist review methodology was utilised and focused on all types of school-based interventions and study designs. A Youth Advisory Group were part of the research team. Multiple search strategies were used to locate relevant evidence. Studies were subject to inclusion criteria and quality appraisal, and included studies were synthesised to produce a programme theory. Seventeen studies were included in the review.

**Results:**

Eight intervention components were necessary to address LGBTQ+ pupils mental health: affirmative visual displays; external signposting to LGBTQ+ support; stand-alone input; school-based LGBTQ support groups; curriculum-based delivery; staff training; inclusion policies; trusted adult. Few school-based interventions for this population group were identified.

**Conclusions:**

The programme theory indicates that “to work” school-based interventions must have a “whole-school” approach that addresses specifically the dominant cis-heteronormative school environment and hence the marginalisation, silence, and victimisation that LGBTQ+ pupils can experience.

## 1. Introduction

There are considerable mental health inequalities between lesbian, gay, bisexual, trans and queer/questioning (LGBTQ+)^1^ young people and their cisgender heterosexual peers (McDermott et al., 2018). Systematic reviews and meta-analyses consistently report a higher prevalence of depression, self-harm, suicidal ideation and attempts, and problematic substance use in LGBTQ+ youth compared to cis-heterosexual youth (Coker et al., 2010; Haas et al., 2011; Irish et al., 2019; Marshal et al., 2011; Saewyc, 2011). A recent meta-analysis of studies comparing suicidality in youth found that compared to cisgender and heterosexual youth, trans youth were six times, bisexual youth five times and lesbian and gay (LG) youth four times more likely to report a history of attempted suicide (Di Giacomo et al., 2018). The restrictions due to Covid-19 have also had a disproportionate impact on LGBTQ+ young people’s mental health (Kamal et al., 2021), and trans and gender diverse youth have been more effected by the pandemic than cis youth (Hawke et al., 2021).

School climate is a leading predictor of students’ emotional and behavioural outcomes (Maxwell et al., 2017). School climate encompasses all elements of the school experience for young people, including quality of teaching and learning, school community relationships, school organisation, and the institutional and structural features of a school environment. This contributes to the quality of interactions for students, teachers and parents, and reflects the norms, values and goals, which represent the educational and social objectives of the school (Council, 2007). School climate affects students’ adaptive psychosocial adjustment (Brand et al., 2008), mental health outcomes (Brand et al., 2003; Roeser et al., 2000), and self-esteem (Hudson-Sharp & Metcalf, 2016). Further, it has been specifically associated with LGBTQ+ youth’s experience of mental health problems, such as depression and suicidality (Marraccini et al., 2022; Peter et al., 2016). A scoping review found that peer victimisation, bullying and safety concerns are prevalent for transgender young people within the secondary schooling environment and these three factors are related to negative mental health outcomes (Mackie et al., 2021). Importantly, a recent systematic review found that positive school climate reduced the risk of suicide and depressive symptoms for LGBTQ students compared to those not in a positive school climate (Ancheta et al., 2021).

The 2016 UNESCO *Out in the Open* (UNESCO, 2016) report identified homophobic, biphobic, and transphobic (HBT) violence in schools as a global problem, and young people who are perceived not to conform to prevailing sexual and gender norms, including those who are LGBTQ+ identified, as most vulnerable. The report concluded that a “comprehensive approach” that involves the whole education sector was the most effective means of addressing the issue. However, only three countries had conducted a large-scale formal evaluation of interventions to tackle HBT violence in schools: the Netherlands, USA, and the UK. In the UK the intervention tackling HBT bullying in schools (Mitchell et al., 2014; Mitchell et al., 2016) has been evaluated nationally (Mitchell et al., 2016). This research remains one of the only evaluations of an intervention that addresses this issue (nationally and globally). A key finding from the evaluation was that for anti-HBT bullying initiatives to be successful they needed to be part of a “whole school” approach (Aiden et al., 2013). The “whole school” approach to HBT bullying involves the entire school rather than being limited to one area of activity (Mitchell et al., 2016). This means moving away from individually oriented approaches that focus on punishing single actors (e.g. bullies), and instead moves to, for example, creating more inclusive curricula and more positive LGBTQ+ representation. Formby (2015) argues that a narrow focus on HBT bullying does not address the multi-levelled approach needed to challenge the dominant cis-heteronormative culture of schools and create an LGBTQ+ inclusive environment. National policy both in the UK and US has tended to have a limited focus on HBT-bullying in schools that concentrates on individual actors and conceals the systemic and pervasive gender and sexual minority inequalities of the wider school climate (Gilbert et al., 2018; Ringrose & Renold, 2010).

Significantly, the UK evaluation of the “whole-school” intervention to tackle HBT-bullying in schools did not evaluate the impact of the intervention on pupil mental health (Mitchell et al., 2016). A systematic review conducted on the impacts of school climate and safe school interventions/programmes on LGBTQ+ youth outcomes (Black et al., 2012) indicated that effective interventions could be grouped according to three common themes: (1) Inclusive programs including support groups (e.g. gay-straight alliances), teacher training programs, and community or student diversity training; (2) Policies encompassing anti-discrimination policies for students and faculty, and clear and enforceable anti-harassment policies; (3) A supportive environment constituting of safe school policies, school board support of inclusive curriculum, available supportive staff, inclusive class discussions about LGBTQ+ issues, and peer acceptance. The authors conclude that safe schools with unclear or unstable policy and programs resulted in more student mental health problems e.g. depression, anxiety (Black et al., 2012).

However, what is clear from this systematic review is that the evidence is sparse and based mainly in US and Canada. More importantly, the explanations for *why* these interventions may improve LGBTQ+ youth mental health have been approximated rather than investigated robustly. This has repercussions for developing interventions to tackle school climate because we do not know what works, for whom, and in what context. That is, we do not understand the mechanisms that underpin the impact of school environment on LGBTQ+ youth mental health or their relationship to different contexts, and this restricts the transferability of interventions to a variety of educational contexts. Taking a public health approach to address this paucity of evidence, this realist review aimed to develop a theoretical understanding of the processes through which interventions within educational settings may prevent or reduce LGBTQ+ adolescents mental health inequalities. The review had two research questions: (1) What evidence exists that school-based interventions can prevent or reduce mental health problems in LGBTQ+ adolescents? (2) How, why, for whom and in what context may school-based interventions prevent or reduce mental health problems in LGBTQ+ adolescents?

## 2. Methods

The realist review method is a theory driven approach to systematic reviews that has been developed for complex social interventions where the experimental paradigm of the randomised control trial may be an inappropriate methodology for producing evidence (Pawson et al., 2005, Greenhalgh et al., 2011). The realist review method uses relevant theory, causal models, and empirical data to establish how a public health intervention causes particular outcomes in what circumstances and for whom. Therefore, a realist review methodology is used to explain why interventions have failed, as well as why they are successful; it provides theoretical explanations for “what works, for whom, under what circumstances, how and why?” (Wong et al., 2013).

The realist research tradition is premised upon an ontological perspective that social reality is interpretative and social actors (human beings) evaluate social reality (Pawson et al., 2005). From a realist perspective, to comprehend how interventions produce outcomes, the role of external reality and human action need to be included within research designs (Greenhalgh et al., 2011). A realist approach attempts to identify the underlying theories that explain the patterns in the way individuals make similar choices within particular intervention conditions (demi-regularities). In other words, a realist perspective identifies the context within which mechanisms (underlying processes) generate the outcomes of interest (e.g. mental health).

The realist review method is appropriate when considering complex interventions which consist of many components and are contingent upon the behaviours and choices of those they seek to benefit. In addition, this method is used when there is little evidence on the effectiveness or acceptability of interventions (Coryn et al., 2011) and/or the literature is heterogenic (Harden et al., 2015; Petticrew, 2015). The realist review methodology follows the same rigorous format as aggregative systematic reviews but the synthesis of the research is focussed on theory generation and specifically aims to broaden understanding of particular interventions rather than testing causal hypotheses (Booth et al., 2011). The key analytic process in a realist review involves the development of a programme theory that explains how and why and in what context the intervention will produce its desired objectives. We followed the establish seven-step realist review protocol (Greenhalgh et al., 2011; Pawson et al., 2005) and publication standards (Wong et al., 2013). The review protocol was registered with PROSPERO (McDermott et al., 2019).

### 2.1. Scoping the literature

The aim of the scoping literature search was to assess the available published literature on school-based interventions aimed at preventing LGBTQ+ youth mental health problems. The results indicated a sufficient evidence base, highlighting specific features consistently contributing to a positive school climate for LGBTQ+ youth such as anti-discrimination policies (Kull et al., 2016; Swanson & Gettinger, 2016; Szalacha, 2003), gay-straight alliances (McGuire et al., 2010; Peter et al., 2016; Szalacha, 2003), LGBTQ+ inclusive curriculum (Snapp et al., 2015), and peer and teacher support in schools (Kohoulat et al., 2015; Peter et al., 2016).

### 2.2. Developing the initial theory

Emmel et al. (2018) highlight the importance of identifying the right theories at the start of a realist project to frame the study. Public mental health interventions are complex and there is a plethora of possible theories. At the start of the review we identified, through expertise in the research team, six existing Middle Range Theories (MRTs) (Jamal et al., 2015) that explain why there is elevated poor mental health in LGBTQ+ populations. These theories were from a range of disciplines and therefore had a variety of perspectives these were: Johnson (2015) psychosocial; Meyer, Hatzenbuehler and Riggs & Treharne all psychology (Hatzenbuehler, 2009; Meyer, 2003; Riggs & Treharne, 2017); Cover and McDermott & Roen both queer theory (Cover, 2016; McDermott & Roen, 2016). In addition, the initial theory was informed by the two UK national reports on the “whole-school approach” to tackling HBT bullying in schools (Mitchell et al., 2014; Mitchell et al., 2016). This intervention was delivered to over 900 schools in the UK. The whole-school approach involves seven identified programme elements: affirmative visual displays; external signposting to LGBTQ+ support; stand-alone input; school-based LGBTQ support groups; curriculum-based delivery; staff training; and inclusion policies. The evaluation highlighted that these seven programme elements were important to ensuring a whole*-*school approach to tackling anti-HBT bullying.

**Figure 1.**
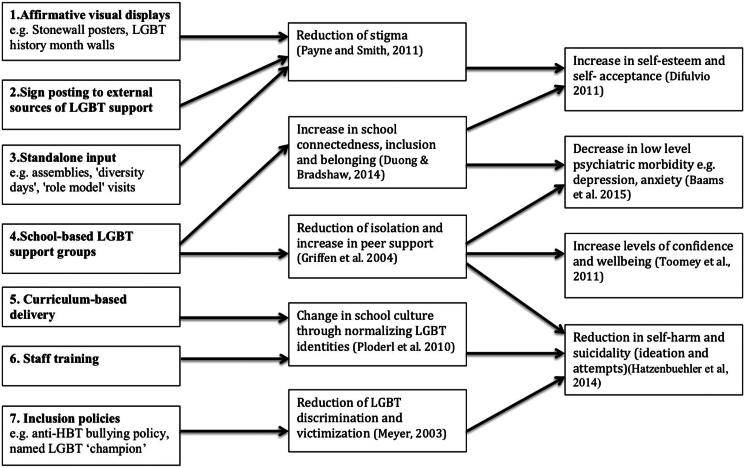
Initial theory.

Close stakeholder engagement from the outset is recommended in realist reviews (Cooper et al., 2017) and five young LGBTQ+ people (aged between 15 and 22) were recruited to participate in a Youth Advisory Group to support the research. The aim of involving LGBTQ+ young people was to help to develop the initial theory and ensure this was informed by stakeholder perspectives. The young people were treated as full members of the research team and were employed by the University as paid researchers. To develop the initial theory, LGBTQ+ young people attended focus groups, and vignettes were used to explore why (and in what context) particular school-based interventions prevent or reduce mental health problems in LGBTQ+ adolescents. Figure 1 outlines the Initial Theory developed from the Middle Range Theories, LGBTQ+ Youth Advisory Group, the empirical papers from the scoping review and the two national reports of the UK anti-HBT bullying intervention. This Initial Theory was then used to inform the realist review with the expectation that it would be refined via the review synthesis process to produce a Programme Theory.

### 2.3. Search strategy

Multiple search strategies were used to locate relevant evidence (including grey literature) from 1999 to March 2021. Good practice in realist reviews characterise searching for literature as an iterative process (Pawson et al., 2005; Rycroft-Malone et al., 2012). Researchers involved in the search process need to be open to the possibility of revisiting searches as theoretical understanding of the interventions becomes more refined. In common with good practice in realist reviews, the search for evidence ended once theoretical saturation had been achieved (Pawson et al., 2005). This involved the research team checking whether newly identified evidence made a significant contribution to the programme theory development process.

The start date of the review was January 1999 to reflect the greater global liberalisation in legislation, policy, and practice regarding LGBTQ+ populations that has taken place over the last two decades (Weeks, 2007). The social and cultural context of LGBTQ+ people’s lives in the West has changed dramatically in the last 20 years (Weeks, 2007) and therefore research before this “modern” era is likely to be less useful to developing a theoretical understanding of the processes through which interventions within contemporary educational settings may prevent or reduce LGBTQ+ adolescents mental health inequalities. For example in the UK there was legislation in place that prohibited schools from discussing sexual orientation within an education setting from 1988 to 2003 (Ellis, 2007) thus preventing interventions to improve LGBTQ+ young people’s school experience. Systematic electronic searches were conducted for peer-reviewed articles published in both discipline specific databases (PubMed, PsycINFO, Embase, ERIC, CINAHL), and multidisciplinary databases (Web of Science, Google scholar, Scopus). Expert consultation, forward citation tracking, and hand-searching were utilised to supplement the electronic database. The search terms were divided into five main domain categories: (i) sexual orientation and gender identity; (ii) age; (iii) education; (iv) mental health and wellbeing; (v) intervention (see supplemental Appendix 1 for full search details).

We conducted targeted searches of the grey literature where there might be relevant evidence. We searched using key organisational websites: Stonewall, Educate & Celebrate, Government Equality Office, Schools Out, The Proud Trust, Government departments in UK, Canada, and USA. In addition, we used Google to search for literature. Given the high number of hits returned we used Google’s relevancy rankings and set a limit of screening the first ten pages of results. This search was also facilitated by contacting subject experts known in the field and identified through our iterative searching.

### 2.4. Inclusion and exclusion criteria

Studies were eligible for inclusion if they were published in English and evaluated: (i) changes to the educational setting; (ii) the effect of the changes on mental health outcomes; (iii) LGBTQ+ young people (see Table 1). Young people aged 10–18 years were included, as this review focussed on adolescents, and there is a paucity of research on these types of intervention amongst younger age groups. The mental health outcomes included direct measures of mental health e.g. anxiety, depression and suicidality, measures associated with mental health e.g. self-esteem, and measures that are important to mental resilience & wellbeing e.g. coping, agency, affect regulation. The inclusion/exclusion criteria were applied by four researchers for their eligibility in the review. Multiple research team members met regularly to discuss the application of the criteria, and whether literature could contribute to building theories about educational interventions and how they may impact LGBTQ+ youth mental health (Pawson et al., 2005).

**Table 1.** Inclusion/exclusion criteria.

	Inclusion	Exclusion
Population	LGBTQ+ youth aged between 10 and 18 years old	Non-LGBTQ+ youth LGBTQ+ youth under 10 years old LGBTQ+ youth aged 19 years and over
Intervention	Measured changes to formal & statutory educational environment Interventions within formal & statutory educational settings	Interventions delivered in non-formal & non-statutory educational settings e.g. youth groups, sports teams
Study design	All empirical study designs Relevant theory-based papers	Papers not empirical based or theory relevant Prevalence & observational studies where no intervention
Outcomes	Poor mental health e.g. anxiety, suicidality, depression Positive mental health including wellbeing, resilience, life satisfaction Psychological factors asso­ciated with mental health e.g. self-esteem, coping, affect regulation Outcomes identified through self-report or standardized assessment	No mental health outcomes reported
Language	English	Any language other than English.
Time frame	Published from 1999	Papers or document published before 1999

### 2.5. Quality appraisal

The appraisal of quality in realist reviews is conducted differently from conventional systematic reviews (Pawson et al., 2005). This is due to the fact that realist review methodology rejects the “hierarchy of evidence” with the randomised controlled trial (RCT) sitting at the top because RCTs focus on what works, and largely overlook the circumstantial factors influencing the effectiveness of an intervention (Pawson, 2006; Pawson et al., 2005). Realist appraisal of studies uses two main criteria to decide whether the study will be considered in the synthesis: relevance and rigour (Pawson et al., 2005).

Firstly, studies were assessed on their relevance. Studies were deemed eligible if they could contribute to building theories about educational interventions and how they may widen or reduce mental health inequalities amongst LGBTQ+ youth. Secondly, the methodological rigour of each individual study was judged to ascertain whether it was trustworthy and had sufficient weight to make a credible contribution to developing the intervention theory. We adapted a previously used realist review methodological appraisal criteria (Jagosh et al., 2011) that asked four main questions: (1) Does the full-text paper describe the research setting? (2) Does the full-text paper indicate empirical research (i.e. that there is some description of methodology, methods, data collection and analysis)? (3) Does the full-text paper describe LGBTQ+ youth mental health outcome data? (4) Does the full-text paper describe an LGBTQ+ youth mental health intervention in an educational setting? These criteria were applied by four of the authors with regular meetings to discuss decisions.

### 2.6. Data extraction

There is no fixed method of data extraction in realist reviews and Wong et al. (2013) suggest that an eclectic and iterative approach to data extraction be adopted. We designed a data extract template and for each included study we extracted the study details, intervention type, context, mechanism, outcome. The data was extracted in “conversation” with the Initial Theory (Figure 1) (Dalkin et al., 2015) and we recorded reflective notes on the data extraction process in relation to the main research question: How, why, for whom and in what context may school-based interventions prevent or reduce mental health problems in LGBTQ+ adolescents?

### 2.7. Data synthesis

The aim of the data synthesis was to refine the Initial Theory through realist synthesis methods to produce a Programme Theory. Early publications describing the realist review process contain little guidance about the synthesis stage (Rycroft-Malone et al., 2012) and more recent publications suggest that any appropriately selected and justified analytic techniques might be used (Wong et al., 2013). We have therefore drawn on Rycroft-Malone et al’s (2012) methodological steps in developing a programme theory and Dalkin et al. (2015) disaggregation of the concept of mechanism into two parts: (i) intervention resources; (ii) participants’ reasoning.

A fundamental building block of realist explanation and developing a programme theory is generating causal links between context, mechanism, and outcome (CMOs). CMOs are theories of causality, a realist approach argues that we cannot observe causal patterns alone and theory (mechanisms) explain the patterns and why there is a relationship. We tabulated the CMOs for each of the included papers, and then integrated these with the Initial Theory we had developed. We used the six identified MRTs (Cover, 2016; Hatzenbuehler, 2009; Johnson, 2015; McDermott & Roen, 2016; Meyer, 2003; Riggs & Treharne, 2017) to develop the causal pathways, these theories were used to deepen the explanations of why each of the intervention components might reduce poor mental health in LGBTQ+ young people. We also ran a workshop with the LGBTQ+ Youth Advisory Group to enhance this thinking. Through the identification of potential CMOs we then refined the Initial Theory to produce a programme theory about why school-based interventions may work to reduce LGBTQ+ adolescents mental health inequalities.

## 3. Results

### 3.1. Search results

**Figure 2.**
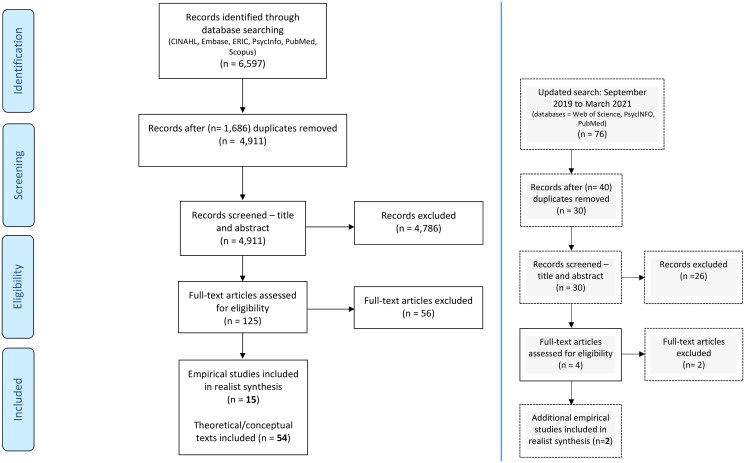
PRISMA diagram showing numbers of papers identified through searching.

The results of the searches, screening and appraisal process are displayed in the PRISMA flow chart (Figure 2). The search criteria applied in electronic databases returned *n* = 6597 results, which after duplicates were removed left *n* = 4911 potentially eligible articles. After title and abstract screening, there were *n* = 125 articles which were included in the next stage. The full-text screening, appraisal, and expert review process eliminated *n* = 56 articles in total, and resulted in *n* = 69 relevant articles, *n* = 15 of which were empirical papers included in the final realist synthesis.

Delays due to Covid-19 restrictions meant that in March 2021, additional searches were run using Web of Science, PsycINFO and PubMed databases (September 2019 to March 2021). These searches returned *n* = 76 results, which after duplicates were removed left *n* = 30 articles. After title and abstract screening, there were *n* = 4 articles which were screened at full-text with expert review, and consequently *n* = 2 additional empirical papers were included in the final realist synthesis. The total number of papers included from the two searches were *n* = 17 (see Table 2).

**Table 2.** Included empirical literature.

Included empirical literature (*n = 17)*
Bopp et al., 2004 (53) Burk et al., 2018 (54) Craig et al., 2014 (55) Goodenow et al., 2006 (56) Gunderson et al., 2021 (57) Hatzenbuehler & Keyes, 2013 (58) Hatzenbuehler et al., 2014 (59) Heck, 2013 (60) Heck, 2015 (61) Lapointe & Crooks, 2018 (62) Lindquist, 2016 (63) Poteat et al., 2015 (64) Poteat et al., 2016 (65) Poteaet et al., 2021 (66) Saewyc et al., 2014 (67) Sandfort et al., 2010 (68) Zhang et al., 2018 (69)

### 3.2. Study characteristics

The majority of the 17 empirical studies included (*n* = 14) were published from 2013 onwards, indicating a growing field of research. None of the studies were conducted in the UK, with only one study (Netherlands) based outside of North America (USA *n* = 12; Canada *n* = 4). The most frequently considered sample characteristics were sexuality, age (range 12–20 years), gender, and ethnicity e.g. Latinx, Pacific Island and First Nations peoples, but Asian ethnicities were less represented. Most included studies involved survey questionnaires (*n* = 12), there were few qualitative designs (*n* = 2), mixed methods (*n* = 1), and pilot programmes (*n* = 2). However, most of these studies also drew on very large sample sizes (e.g. 55,599), with data gathered using validated tools.

The interventions most frequently considered in the included studies were Gay-Straight Alliances (*n* = 7), followed by health-based programmes (*n* = 4). Some studies explored GSAs in isolation (*n* = 3), whilst other interventions considered school policies and climate (*n* = 2), as well a film-based intervention (*n* = 1). The most frequently considered mental health outcome in the included studies was suicidality (*n* = 8), other outcomes were self-esteem (*n* = 3) and coping (*n* = 2). Wellbeing, depression and anxiety, purpose in life, mastery, and sense of agency were also measured in some studies (see supplemental Appendix 2 for included studies characteristics).

### 3.3. Programme theory

The final programme theory detailing the context-mechanism-outcome configurations for school-based interventions is outlined in Table 3. Explaining why and how an intervention may have an impact on mental health is complex (and under-researched). To help make explicit and theorise “what changes” and “how it changes” as a result of an intervention, we developed Dalkin’s (2015) dual concept of mechanism – intervention resource and response mechanism – and added a new third mechanism factor which we called the “vector of change.” The inclusion of the new concept of “vector of change” was to capture the multiple causal pathways at which a mental health intervention may work e.g. psychological, behavioural, emotional, environmental, social.

**Table 3.** Programme theory.

CONTEXT	MECHANISM 1 Intervention Resources	MECHANISM 2 Vector of change What changes?	MECHANISM 3 Cognition How does it change?	OUTCOME
School culture (gender and sexuality) LGBTQ++ Champion Legal, Policy & Economic Directives	**1. Affirmative displays** **2. External signposting** (Hatzenbuehler, Birkett, Wagenen, & Meyer, 2014; Heck, 2013) **3. Stand alone** **input** (Burk, Park & Saewyc, 2018) **4. Support Groups** (Bopp, Juday & Charters, 2004; Craig, Austin & McInroy, 2014; Goodenow, Szalacha, & Westheimer, 2006; Gunderson et al., 2021; Hatzenbuehler, Birkett, Wagenen, & Meyer, 2014; Heck, 2013; Heck, 2015; Lapointe & Crooks, 2018; Lindquist, 2016; Poteat et al., 2015; Poteat, Yoshikawa & Calzo, 2016; Poteat et al., 2021; Saewyc et al., 2014) **5. Curriculum** (Gunderson et al., 2021; Hatzenbuehler, Birkett, Wagenen, & Meyer, 2014; Lindquist, 2016) **6. Staff training** (Goodenow, Szalacha, & Westheimer, 2006; Gunderson et al., 2021; Hatzenbuehler, Birkett, Wagenen, & Meyer, 2014) **7. Inclusion policies** (Goodenow, Szalacha, & Westheimer, 2006; Gunderson et al., 2021; Hatzenbuehler & Keyes, 2013; Hatzenbuehler, Birkett, Wagenen, & Meyer, 2014; Lindquist, 2016; Saewyc et al., 2014) **8. Talking to trusted adult** (Bopp, Juday & Charters, 2004; Craig, Austin & McInroy, 2014; Goodenow, Szalacha, & Westheimer, 2006; Gunderson et al., 2021; Lindquist, 2016; Zhang et al., 2020)	**1. School culture & environment** (Goodenow, Szalacha, & Westheimer, 2006; Gunderson et al., 2021; Hatzenbuehler, Birkett, Wagenen, & Meyer, 2014; Lindquist, 2016; Sandfort et al., 2010) **2. Relationships & interactions** (Bopp, Juday & Charters, 2004; Burk, Park & Saewyc, 2018; Goodenow, Szalacha, & Westheimer, 2006; Lapointe & Crooks, 2018; Lindquist, 2016) **3. Behaviours & actions** (Bopp, Juday & Charters, 2004; Goodenow, Szalacha, & Westheimer, 2006; Gunderson et al., 2021; Hatzenbuehler & Keyes, 2013; Hatzenbuehler, Birkett, Wagenen, & Meyer, 2014; Heck, 2013; Heck, 2015; Poteat, Yoshikawa & Calzo, 2016; Saewyc et al., 2014; Zhang et al., 2020) **4. Self, thoughts, beliefs** (Bopp, Juday & Charters, 2004; Craig, Austin & McInroy, 2014; Lapointe & Crooks, 2018; Poteat et al., 2015) **5. Affect, feeling, emotions** (Bopp, Juday & Charters, 2004; Burk, Park & Saewyc, 2018; Craig, Austin & McInroy, 2014; Goodenow, Szalacha, & Westheimer, 2006; Gunderson et al., 2021; Hatzenbuehler & Keyes, 2013; Hatzenbuehler, Birkett, Wagenen, & Meyer, 2014; Heck, 2015; Lapointe & Crooks, 2018; Lindquist, 2016; Poteat et al., 2015; Poteat et al., 2021; Saewyc et al., 2014; Zhang et al., 2020)	**Affirmation (I am good)** (Craig, Austin & McInroy, 2014; Lapointe & Crooks, 2018) **Agency (I can do**) (Bopp, Juday & Charters, 2004; Poteat, Yoshikawa & Calzo, 2016) **Advocacy (I can make better)** (Poteat, Yoshikawa & Calzo, 2016) **Belonging (I am included)** (Bopp, Juday & Charters, 2004; Lindquist, 2016) **Connectedness (I am like you)** (Burk, Park & Saewyc, 2018) **Coping (I am positive)** (Bopp, Juday & Charters, 2004; Craig, Austin & McInroy, 2014; Lapointe & Crooks, 2018) **Recognition (I count)** **Safety (I feel no fear)** **Usualising (I am accepted)** (Bopp, Juday & Charters, 2004)	**Decrease in poor MH** (Bopp, Juday & Charters, 2004; Burk, Park & Saewyc, 2018; Craig, Austin & McInroy, 2014; Goodenow, Szalacha, & Westheimer, 2006; Gunderson et al., 2021; Hatzenbuehler & Keyes, 2013; Hatzenbuehler, Birkett, Wagenen, & Meyer, 2014; Lindquist, 2016; Poteat et al., 2015; Saewyc et al., 2014; Sandfort et al., 2010; Zhang et al., 2020) **Increase in factors associated with + MH** (Goodenow, Szalacha, & Westheimer, 2006; Heck, 2015; Lapointe & Crooks, 2018; Poteat, Yoshikawa & Calzo, 2016; Poteat et al., 2021)

It is important to note that the Programme Theory was developed as a “whole-school approach” but the context in which the intervention was delivered was often under-reported and unexamined in the included empirical studies. We found only three key contextual factors in the included studies that were important to the success of school-based interventions that improved LGBTQ+ young people’s mental health. Firstly, a positive baseline (before intervention delivery) school culture towards sexual and gender diversity was highlighted. Secondly, the presence of a member of education staff or pupil who “championed” LGBTQ+ pupil equality and worked toward achieving a LGBTQ+ inclusive environment. Thirdly, where interventions were implemented in schools with national legal, policy and economic directives that specifically addressed LGBTQ+ equality, they were more likely to be successful in improving LGBTQ+ pupil mental health outcomes.

Mechanism 1 in the Programme Theory (Table 3) focused on the intervention resources and contained eight components that aimed to reduce LGBTQ+ adolescent poor mental health burden. The original Initial Theory contained seven components, we have added an eighth component, “a trusted adult to talk to,” that the included studies suggested was important to reducing poor mental health. Across the literature we reviewed, there was evidence that a combination of these components was most likely to be successful in reducing the poor mental health of LGBTQ+ young people.

Mechanism 2 “vectors of change” in the Programme Theory addresses the multiple causal pathways (psychological, behavioural, emotional, environmental, social) at which a particular mental health intervention component may work. We identified five vectors of change through which the intervention component may work: the school environment and culture becoming more LGBTQ+ inclusive; relationships and interactions between individuals (both pupil to pupil and staff and pupil) becoming less hostile and more connected; individual behaviours and actions changing; individual LGBTQ+ pupils thoughts and beliefs about the selfimproving e.g. self-esteem, positive identity orientation; individual affect, feelings & emotions becoming less problematic. The literature suggested that these five vectors of change may co-occur.

Mechanism 3 “cognition” or “response mechanism” explains how the intervention effects the individual actor’s reasoning and response to intervention components. Here we have identified the importance of “affirmation” of LGBTQ+ identities to improving self-esteem, self-acceptance, confidence, and validation. “Agency” as a capacity to initiate and sustain actions regarding LGBTQ+ status, and “Advocacy” as a process of empowerment to change school culture. “Belonging” to the school and feeling included was an important factor in good mental health for LGBTQ+ pupils especially in reducing isolation and loneliness. Relatedly, school connectedness and feeling similar to others (peers and staff) improved mental health. “Coping” this was both individual coping with hostility, invisibility or misgendering but also includes group problem solving. “Recognition” of LGBTQ+ identity, the synthesis suggested, was important to validating LGBTQ+ young people within school. Feeling “safe” and without fear was crucial to improving LGBTQ+ youth mental health. The final mechanism “usualising” was the feeling that being LGBTQ+ was accepted and “normal” in school culture. In our theoretical development, it was evident that these cognitive processes would co-occur and be inter-related.

Overall, the evidence suggested that interventions that seek to make the school environment more inclusive for LGBTQ+ young people can improve mental health outcomes by both reducing poor mental health e.g. suicidal feelings and depression, and increasing factors associated with good mental health e.g. self-esteem and coping. However, the lack of evidence on the contextual factors in which interventions are successfully delivered limits our understanding of how inclusivity in schools can be promoted. The Programme Theory we have developed attempts to elucidate the underpinning mechanisms that explain how and why these interventions may work to improve LGBTQ+ youth mental health.

## 4. Discussion

This realist review has developed a programme theory of how, why, for whom and in what context school-based interventions prevent or reduce mental health problems in LGBTQ+ adolescents. To improve the mental health of LGBTQ+ pupils i.e. for the intervention to “work” school-based interventions must address specifically the marginalisation, silence, victimisation, and misrecognition that LGBTQ+ pupils experience within the dominant cis-heteronormative school environment. The pervasiveness of learning in a school culture that excludes pupils whose sexual and gender is at odds with the dominant norms was noted by many scholars whose work contributed to our review. The literature we engaged with suggested that to change the school environment to improve LGBTQ+ pupils mental health requires a multi-level approach (Hatzenbuehler, 2009; Saewyc, 2011) or in the UK, this has been termed the whole-school approach to LGBTQ+ school inclusivity (Formby, 2015; Mitchell et al., 2016; UNESCO, 2016).

The strength of the whole-school approach is that the intervention aims to change the school environment at the structural, cultural, and individual level. This is because hostility, discrimination and bullying on the basis of perceived gender and sexual diversity does not arise purely from individuals or groups but rather reflects (and upholds) cis-heteronormativity (dominant norms surrounding gender as a binary and heterosexuality). Developing a LGBTQ+ inclusive school environment needs policies that specifically tackle HBT bullying and discrimination (in staff and pupils), and policies that promote equality across gender and sexual diversity. It includes staff training and support for implementing such policies and an inclusive curriculum, providing school resources for LGBTQ+ support and activism (along with allies) and ensuring there are school staff that LGBTQ+ pupils can talk to and trust. This all creates better relationships in school between staff and pupils where trust, care, belonging, connection, and confidence can flourish, and mental health can improve.

Across the literature we reviewed, scholars worldwide were struggling with similar difficulties in trying to understand the problem and provide solutions. The background for each paper was the marginalisation and stigmatisation of young LGBTQ+ lives, the impact that school life had on their mental health, and the dearth of appropriate school-based interventions aimed at generating an inclusive environment and improving mental health. Often, scholars noted the absence of policy attention to the matter of LGBTQ+ pupils’ difficulties in schools. We need more research and datasets that can examine how best to address the school mental health of LGBTQ+ pupils. Our realist review exposed the limited evidence of the various contextual factors that may be important to the success of interventions. For example, does the ethnic composition of the school matter (probably yes)? Does religion? Or socioeconomic status? How should interventions be delivered and implemented? When and by who? Research of this nature requires both large datasets that better measure interventions and different mental health outcomes, but we also need qualitative research that explores how and why school culture can change, and the contextual factors that impact on the effectiveness of interventions.

The strength of our realist review is that it provides a baseline theoretical understanding for why and how school-based interventions might improve mental health for LGBTQ+ young people. Much of the literature we reviewed had limited data on how the interventions may or may not work, with some notable exceptions such as Saewyc (2011). A further strength of the Programme Theory is that it is drawn from a synthesis of empirical data from a range of disciplines (psychology, public health, sociology, social work), a national evaluation of a UK anti-bullying in schools intervention, theories that explain LGBTQ+ youth mental health disparities and the direct perspectives of LGBTQ+ young people.

However, the review is limited partially by the limitations of the empirical studies we were able to locate (we acknowledge our inclusion/exclusion criteria was limited by language). Often scholars where relying on already existing data and “matching” this with created variables. Consequently, there was a variation in the demographic characteristics reported, the terms used, and how different groups are categorised. In addition, the included studies lacked an intersectional perspective on LGBTQ+ identities; particularly attention on the variation between lesbian, gay, bisexual, transgender, and diverse gender identities, as well as other intersectional characteristics, such as ethnicity, poverty, rurality, and disability. The use of pre-existing large-scale surveys introduces a restrictive dynamic to the questions and ideas explored by the studies, potentially limiting the granularity of detail available for a realist review. This also explains why there was a dearth of research on the contextual factors in which interventions can be successfully delivered. As a consequence, there was not adequate evidence to fully plot the causal pathways and detail the generative causal connections. Realist reviews also have methodological limitations that arise from the complexity of the theory-orientated method. It is impossible to record every single decision, and a realist approach does not comply with the “reproducibility principle” (Pawson et al., 2004). Consequently, realise reviews deliver tentative and fallible findings, and there is “no certitude” in terms of recommendations (Pawson et al., 2004).

Despite these limitations, this realist review provides the first baseline theoretical understanding for why and how school-based interventions might improve mental health for LGBTQ+ young people. This is a starting point; it should now be developed through empirical refinement to improve understanding of the contextual factors in which successful school-based interventions are delivered, and the causal pathways that reduce LGBTQ+ pupil poor mental health. Researchers should also endeavour to explore early and preventative school-based interventions amongst younger LGBTQ+ groups.

## Supplementary Material

Supplemental Material

## Notes

1 We use LGBTQ+ to refer collectively to sexual minority and gender diverse identities because of the proliferation of terms used by young people. References to other research use the author’s original terminology for sexuality/gender.
