# Supplementary material for: Reducing LGBTQ+ adolescent mental health inequalities: a realist review of school-based interventions
Source: J Ment Health. 2023 Aug 17;33(6):768–78. doi: 10.1080/09638237.2023.2245894 (PMC11789706; doi:10.1080/09638237.2023.2245894)
Supplement: Supplemental Material [file IJMH_A_2245894_SM1573.zip › cjmh-2023-0215-File007.docx]

Appendix 1: Search terms for electronic database search

| Sexual orientation and gender identity domain  “LGBT*” OR lesbian OR gay OR “bisexual” OR trans* OR transgend* OR “same-sex” OR “sexual minorit*” OR homosexual* OR "sexual orientation" OR queer* OR questioning OR "gender atypicality" OR “gender-atypicality” OR “transsexual*" OR “gender non-binary” OR "sexual identit*"OR non-heterosexual* |
| --- |
| Age domain  Youth OR adolescen* OR teen* OR (young N2 (person OR people* OR adult*)) OR student* OR pupil* OR children ) OR "High School Students" OR "Junior High School Students" OR "Special Education Students" OR "Vocational School Students" |
| Education domain  School* OR education OR academy OR academies* OR college OR (“further education” OR “FE”)) OR “non-curricul*” OR “extra-curricul*” OR “inclusive curricul* OR “after school” OR (school N2 (culture* OR climate OR ethos OR norms OR values)) OR ((school OR education*) AND (policies OR policy)) OR (school N2 (leader* OR governance)) OR “school belong*” OR “school connected*” OR OR “school isolation” OR teachers* OR (teaching N2 (staff OR personnel)) OR (school N2 (staff OR personnel)) OR ((non-teaching OR non teaching) N2 staff) |
| Mental health domain  ( ((mental OR emotional OR psychosocial OR psycho-social) N2 (health OR wellbeing OR well-being OR problem*OR difficult* OR disorder* OR risk*)) OR (((mental OR emotional) AND health) N2 (problem* OR disorder* OR condition*OR difficult* OR risk* OR poor)) OR (behavio?r* N2 (problem* OR disorder* OR difficult* OR risk*)) OR ((emerg* OR “sub-clinical” OR “sub clinical”) AND ((mental OR emotional) AND health) N2 (problem* OR difficult* OR disorder*)) OR depress* OR anxi* OR suicid* OR (“self-harm*” OR “self harm*” OR “self-injur*”) OR stress* OR distress* OR “ADHD” OR “ADD” OR ((externali#ing OR internali#ing) AND (disorder* OR problem*)) OR ((“attention deficit” OR “attention deficit and hyperactivity” OR “oppositional defiant” OR conduct) AND disorder*)) OR resilien* OR autonomy OR (“self acceptance” OR “self-acceptance”) OR (“self-esteem” OR “self esteem”) OR (“self-efficacy” OR “self efficacy”) OR (“self-criticism” OR “self-criticism”) OR hopelessness ) |
| Intervention domain  ( prevent* OR promot* OR ((primary OR secondary OR universal OR targeted OR selective OR indicated) AND prevent*) OR intervention* OR ((crisis OR early OR “school based” OR “school-based” OR education*) N2 (intervention)) OR support OR ((peer OR student OR social) N2 support)) OR reform* OR “improvement strategy*” OR program* OR initiative* OR measure* OR counsel* OR (school N2 counsel*) OR “safe space” ) |
