# Supplementary material for: Reducing LGBTQ+ adolescent mental health inequalities: a realist review of school-based interventions
Source: J Ment Health. 2023 Aug 17;33(6):768–78. doi: 10.1080/09638237.2023.2245894 (PMC11789706; doi:10.1080/09638237.2023.2245894)
Supplement: Supplemental Material [file IJMH_A_2245894_SM1573.zip › cjmh-2023-0215-File008.docx]

| **Reference** | **Study Design** | **Intervention** | **Sample Characteristics** | **Mental Health Outcomes** | **Findings Summary** |
| --- | --- | --- | --- | --- | --- |
| Bopp PJ, Juday TR, Charters CW. A School-Based Program to Improve Life Skills and to Prevent HIV Infection in Multicultural Transgendered Youth in Hawai'i. Journal of Gay & Lesbian Issues in Education. 2004;10;1(4):3-21. (USA) | Survey questionnaire study and semi-structured confidential interviews | Chrysalis is a weekly after-school drop-in group on O’ahu high school campuses for transgendered and questioning youth | n=9 Chrysalis members, n=9demographically matched TG youth, and five key informants. Mean age of Chrysalis members was 16.5 years (SD = 1.24, range = 14-18), and mean age of non-Chrysalis TG youth in the comparison group was 15.8 years (SD = 1.30, range = 13-17). Most of the individuals in both groups were multi-ethnic, and all were of Pacific Islander and/or Asian descent. The only individuals from a single ethnic group were Samoans. | The Rosenberg Self-Esteem Scale (Rosenberg, 1965); Suicidality | The Chrysalis group scored better than the comparison group for every objective-related topic, thus providing preliminary evidence of program effectiveness. Positive comments in the interviews from Chrysalis members and key informants further support the program’s success. While causal relationships could not be established because pre-intervention measurements were not taken and due to the small sample sizes, Chrysalis is perceived as playing an important role in improving the educational outcomes and life skills and in preventing HIV/STD among TG youth |
| Burk J, Park M, Saewyc EM. A media-based school intervention to reduce sexual orientation prejudice and its relationship to discrimination, bullying, and the mental health of lesbian, gay, and bisexual adolescents in Western Canada: A population-based evaluation. International journal of environmental research and public health. 2018;15(11):2447. (Canada) | The evaluation mapped data about Out in Schools presentations onto student data from the random cluster-stratified, province-wide 2013 British Columbia Adolescent Health Survey (BCAHS) as well as potential confounding variables of Gay-Straight Alliance clubs (GSAs) and inclusive school policies. | A film-based intervention 'Out in Schools' designed to reduce sexual orientation prejudice and foster inclusive school attitudes | Lesbian, gay, and bisexual (LGB) and heterosexual (HET) students in grades 8 through 12 (ages 13 to 18; unweighted N = 21,075, weighted/scaled N = 184,821). | Suicidality: Suicidal ideation was measured with one binary item: “During the past 12 months, did you ever seriously consider killing yourself (attempting suicide)?” Response options were “yes or “no”. | Out in Schools presentations were associated with reduced odds of LGB students experiencing discrimination, and both LGB and HET girl students being bullied or considering suicide, and increased levels of school connectedness, even after controlling for GSAs and policies. Out in Schools appears to have an additive contribution to reducing orientation prejudice and improving LGB and heterosexual student wellbeing within schools. |
| Craig SL, Austin A, McInroy LB. School-based groups to support multi-ethnic sexual minority youth resiliency: Preliminary effectiveness. Child and adolescent social work journal. 2014;31(1):87-106. (Canada) | Pilot uncontrolled trial | Affirmative supportive safe and empowering talk (ASSET), the first LGBTQ affirmative school-based group counselling intervention created specifically to promote resiliency | Multi-ethnic Sexual Minority Youth (n = 263) lesbian (38 %), bisexual (32 %), gay (22 %); Hispanic all races (74 %), Black, non-Hispanic (21 %), White non-Hispanic (3 %); as well as female (72 %), male (27 %), and transgender (1 %). Ages ranged from 13 to 20 with a mean age of 16.7 years | The Rosenberg Self-Esteem Scale (SES); The Proactive Coping Inventory (PCI) | Post-intervention analysis using general linear modelling suggests that self-esteem and proactive coping increased significantly across all subgroups, while social connectedness remained constant. ASSET holds promise to enhance the resiliency of SMY in school-based practice settings |
| Goodenow C, Szalacha L, Westheimer K. School support groups, other school factors, and the safety of sexual minority adolescents. Psychology in the Schools. 2006;43(5):573-89. (USA) | Survey questionnaire study: This study used data from the Massachusetts Youth Risk Behavior Survey (Massachusetts Department of Education, 2000), matched with school-level data from state records and school principals | Programs and policies: LGB Student Support; Perceived Staff Support; Other Peer-Support Group; Psychological Counselling; Monitoring At-Risk Students; Student Court; Sexual Harassment Training; Community-Service Learning; Peer-Tutoring Program; Anti-Bullying Policy | Of the 56 schools with principal data, 52 included at least 1 MYRBS participant who could be categorized as “sexual minority” based on two MYRBS questions. The first question asked youth which term best described them: heterosexual (straight), gay or lesbian, bisexual, or not sure. The second asked “With whom have you had sexual contact?”: no one, female(s), male(s), both female(s) and male(s). The 202 adolescents who self-identified as LGB and/or reported any same-sex sexual contact were considered “sexual minority” for the purposes of this study | Suicidality (Savin-Williams, 2001); Depression (MYRBS) | As hypothesized, sexual minority adolescents in schools with LGB support groups reported lower rates of victimization and suicide attempts than those in other schools. Victimization and perceived staff support predicted suicidality. Several additional school factors were associated with the safety of sexual minority students |
| Gunderson LM, Shattuck DG, Green AE, Vitous CA, Ramos MM, Willging CE. Amplification of school-based strategies resulting from the application of the dynamic adaptation process to reduce sexual and gender minority youth suicide. Implementation Research and Practice. 2021 Jan;2:2633489520986214. (USA) | Qualitative data derived from 36 semi-structured interviews and 16 focus groups conducted with school professionals | The Expert Recommendations for Implementing Change (ERIC), a taxonomy of discrete implementation strategies used in health care settings, was adapted for schools. The School Implementation Strategies Translating the ERIC Resources (SISTER) resulted in 75 discrete implementation strategies. This paper examined which SISTER strategies were used to implement six evidence-informed practices (EIPs), to reduce suicidality among LGBTQ high school students | 36 administrators and leads participated in individual interviews. 43 team members from 16 teams took part in 16 small focus groups. The sample was: 69.14% white; 76.54% female; 79.01% heterosexual | Suicidality; plus 'other negative outcomes' (depression and bullying). | 20 SISTER strategies were encouraged under the dynamic adaptation process (DAP), nine of which were amplified by school personnel. Nine additional SISTER strategies not specifically built into the DAP were implemented independently by school personnel, given the freedom the DAP provided, resulting in a total of 29 SISTER strategies. This study offers insight into how schools select and elaborate implementation strategies. The DAP fosters freedom to expand beyond study-supported strategies. Findings from this study may be a launching pad for exploring how the use of strategies to address equity issues functions in practice |
| Hatzenbuehler ML, Keyes KM. Inclusive anti-bullying policies and reduced risk of suicide attempts in lesbian and gay youth. Journal of Adolescent Health. 2013; 1;53(1):S21-6. (USA) | Survey questionnaire study | (1) the absence of anti-bullying policies; (2) the presence of anti-bullying policies including specific categories (e.g., gender, race, religion), but not sexual orientation (which are hereafter referred to as “restrictive anti-bullying policies”); and (3) anti-bullying policies that were inclusive of sexual orientation (which are hereafter referred to as “inclusive anti-bullying policies”) | Of the 33,714 original OHT respondents, 30,439 (90.3%) self-identified as heterosexual, 301 (0.9%) self-identified as gay or lesbian, and 1,112 (3.3%) self-identified as bisexual. Participants who indicated that they were “not sure” about their sexual orientation (n=653; 1.9%) were excluded from analyses, consistent with previous studies. An additional 1,209 respondents did not complete the sexual orientation item and were also excluded. Consequently, the final sample size was 31,852 11th grade students | Participants were asked the number of times they attempted suicide during the past 12 months. Given the non-normal distribution, suicide attempts were examined as a dichotomous outcome. The suicide question used in the OHT was based upon a measure from the Youth Risk Behavior Surveillance Survey (YRBS) | Lesbian and gay youths living in counties with fewer school districts with inclusive anti-bullying policies were 2.25 times (95% C.I.: 1.13, 4.49) more likely to have attempted suicide in the past year compared to those living in counties where more districts had these policies. Inclusive anti-bullying policies were significantly associated with a reduced risk for suicide attempts among lesbian and gay youths even after controlling for sociodemographic characteristics (sex, race/ethnicity) and exposure to peer victimization (OR=0.18, 95% CI: 0.03–0.92). In contrast, anti-bullying policies that did not include sexual orientation were not associated with lower suicide attempts among lesbian and gay youths (OR=0.38, 95% CI: 0.02–7.33). Inclusive anti-bullying policies may exert protective effects for the mental health of lesbian and gay youths, including reducing their risk for suicide attempts |
| Hatzenbuehler ML, Birkett M, Van Wagenen A, Meyer IH. Protective school climates and reduced risk for suicide ideation in sexual minority youths. American journal of public health. 2014;104(2):279-86. (USA) | Survey questionnaire study (pooled 2005 and 2007 Youth Risk Behavior Surveillance Surveys from 8 states and cities, and the 2010 School Health Profile Survey, compiled by the Centers for Disease Control and Prevention) | Gay-Straight Alliance and safe spaces for LGBTQ youths; curricula on health matters relevant to LGBTQ youths (e.g., HIV); prohibit harassment based on sexual orientation or gender identity; encourage staff to attend trainings on creating supportive environments for LGBTQ youths, and facilitate access to providers off school property that provide health and other services specifically targeted to LGBTQ youths | Final sample size was n=55,599. Gender, age, race/ethnicity, and sexual orientation were assessed via self-report, but only reported by suicide outcome data | Suicide thoughts; suicide plan; suicide attempt (%, 95% CI) | Lesbian, gay, and bisexual students living in states and cities with more protective school climates reported fewer past-year suicidal thoughts than those living in states and cities with less protective climates (lesbians and gays: odds ratio [OR] = 0.68; 95% confidence interval [CI] = 0.47, 0.99; bisexuals: OR = 0.81; 95% CI = 0.66, 0.99). Results were robust to adjustment for potential state-level confounders. Sexual orientation disparities in suicidal thoughts were nearly eliminated in states and cities with the most protective school climates. School climates that protect sexual minority students may reduce their risk of suicidal thoughts |
| Heck NC. Expanding, refining, and replicating research on high school gay-straight student alliances and sexual minority youth (Doctoral dissertation, University of Montana, 2013). (USA) | Survey questionnaire study | High school GSAs, LGBT community centres and community groups, PFLAG chapters, LGBT college and university student groups | A total of 316 sexual minority youth completed an online survey. Of the 316 participants included in the analytic sample, 54.4% (n = 172) identified as female, 37.7% (n = 119) identified as male, and 7.9% (n = 25) identified as transgender (female to male = 10; male to female = 4) or with another minority gender identity (other gender = 11). The average age of participants was 16.75 years (SD = 0.78); 44.6% (n = 141) of participants were 16 years old, 38.0% (n = 120) were 17 years old, 15.5% (n = 49) were 18 years old, and 1.9% (n = 6) were 19 years old. Approximately 70% (n = 217) of participants identified as Caucasian, while 9.2% (n = 29) identified as Hispanic, Chicano, or Mexican American, 8.5% (n = 27) identified as African American or Black, 4.4% (n = 14) identified as American Indian, Native American, or Alaskan Native, 4.1% (n = 13) identified as Asian American, and 5.1% (n =16) selected “other” to best represent their ethnic or racial background. Finally, 67.4% (n = 213) of participants selected “single” to reflect their relationship status, while 20.9% (n= 66) and 11.7% (n = 37) selected “committed relationship” and “dating” to reflect their relationship statuses | Brief Symptom Inventory (BSI; Derogatis, 1993) and the PTSD Checklist- Civilian version (PCL-C; Elhai, Gray, Kashdan, & Franklin, 2005; Weathers, Litz, Herman, Huska, & Keane, 1993) | Results indicate that youth attending a high school with a GSA reported more favourable substance use outcomes when compared to peers attending a high school without a GSA. However, this association was not present when examining mental health outcomes, which may indicate that GSAs promote favourable mental health outcomes in sexual minority young adults by way of reduced substance use in late adolescence. This association may also be the result of undetected interaction effects or non-linear associations among predictor and outcome variables |
| Heck NC. The potential to promote resilience: Piloting a minority stress-informed, GSA-based, mental health promotion program for LGBTQ youth. Psychology of Sexual Orientation and Gender Diversity. 2015;2(3):225. (USA) | Uncontrolled pilot feasibility and acceptability study | A mental health promotion program that was developed to address minority stressors and promote coping skills among lesbian, gay, bisexual, transgender, and queer (LGBTQ) youth | Ten GSA members participated in the study, and although each session was well attended, attendance was not consistent across all study sessions. Two participants attended four sessions, one attended three sessions, six attended two sessions and one participant attended one session. The modal number of sessions attended was 2, with an average of 2.4 sessions attended per participant. Those identified by the GSA advisor as being consistent in their attendance at meetings attended an average of 2.8 sessions | The first session emphasized the identification of minority and general stressors, which was followed by a discussion of coping strategies. The remaining sessions emphasized the development of cognitive coping, affect regulation, and problem-solving skills. After each session, participants completed a 13-item feedback form. Ten items assessed acceptability and three open-ended items allowed participants to provide constructive feedback | Although the program was feasible to implement within the GSA setting, attendance at the sessions was variable. Those who attended the sessions reported them to be enjoyable, informative, relevant to their lives, and potentially helpful for other LGBTQ students. After revising the program, future research is needed to investigate its dissemination potential and determine whether the program can disrupt the minority stress-psychiatric distress relationship |
| Lapointe A, Crooks C. GSA members' experiences with a structured program to promote well-being. Journal of LGBT Youth. 2018;2;15(4):300-18. (Canada) | Qualitative approach-focus groups | Gender and Sexuality Alliances (GSA)1 are relevant venues to reach LGBT2QC students and deliver healthy relationships and mental health promotion programming at school | Approximately 65 youth from secondary schools.  Moreover, no demographic data was collected from youth (e.g., race, gender identity, sexual orientation, etc.) | Wellbeing | Results indicated that the program helped youth validate and affirm their identities and expressions. The program also afforded youth structured opportunities to identify and process minority stressors, and develop essential coping strategies to bolster their well-being and manage their toxic relationships |
| Lindquist LM. School supports for LGBTQ students: Counteracting the dangers of the closet (Doctoral dissertation, University of Montana, 2016). (USA) | Survey questionnaire study | Thesis is based on survey data | Gay-straight student alliances, inclusive curricula, antidiscrimination policies, supportive school personnel, accepting peers, and safe zones | Depression and anxiety were measured by the short form of the Depression Anxiety Stress Scale (DASS-21; Lovibond & Lovibond, 1995a). Internal consistency was α = .805 overall, α =.868 for depression, and α = .806 for anxiety | Results indicated that identity integration is significantly correlated with depression and school belonging. They also showed that school supports significantly moderate the relationships between identity integration and absenteeism, and between identity integration and school belonging in female-identified students |
| Poteat VP, Yoshikawa H, Calzo JP, Gray ML, DiGiovanni CD, Lipkin A, Mundy‐Shephard A, Perrotti J, Scheer JR, Shaw MP. Contextualizing Gay‐Straight Alliances: Student, advisor, and structural factors related to positive youth development among members. Child development. 2015;86(1):176-93. (USA) | Purposeful sampling of school GSAs in Massachusetts. Surveys gathered from students and advisors. Ethnographic component involving observation of a regularly scheduled GSA meeting. | To examine which components of Gay-Straight Alliances (GSAs) promote wellbeing | 146 youth in grades 9 to 12 who were current GSA members across thirteen Massachusetts high schools. The GSAs ranged in size from four to 35 students (M size = 11 students, SD = 8 students). Students ranged from 14 to 19 years of age (Mage = 16.04, SD = 1.26), represented across grade levels (Grade 9: n = 46; Grade 10: n = 38; Grade 11: n = 33; Grade 12: n = 23). Of the participants, 57 identified as heterosexual, 36 as bisexual, 27 as gay or lesbian, 9 as questioning their sexual orientation, 12 identified as ‘other’, and 5 did not report their sexual orientation. Most students identified as female (n = 99), 40 as male, 2 as transgender (both as female to male), while 4 identified as ‘other’ and 1 did not report their gender. There were 18 advisor participants across the thirteen GSAs | Measures - 7-item Mastery Scale, 12-iten Purpose in Life scale and 10-item Rosenberg Self-Esteem scale | In multilevel models, GSA support predicted all outcomes. Racial/ethnic minority youth reported greater wellbeing, yet lower support. Youth in GSAs whose advisors served longer and perceived more control and were in more supportive school contexts reported healthier outcomes. GSA advocacy also predicted purpose. Ethnographic notes elucidated complex associations and variability in how GSAs operated |
| Poteat VP, Calzo JP, Yoshikawa H. Promoting youth agency through dimensions of gay–straight alliance involvement and conditions that maximize associations. Journal of Youth and Adolescence. 2016;45(7):1438-51. (USA) | Secondary analyses of GSA Network survey. Multivariate analysis of variance for different functions and characteristics of GSAs. Produces multilevel models of youth agency | Gay-Straight Alliances (GSAs) - study examines functions and characteristics of GSAs, and how these contribute to sense of agency in young people (agency considered as a component of wellbeing) | The sample included 295 youth in 33 Massachusetts GSAs (69 % LGBQ, 68 % cisgender female, 68 % white; M = 16.06 years, ranging in age from 13 to 20 years) | Sense of agency measured by six-item State Hope Scale that assesses agency and pathways to achieving goals (Snyder et al. 1996; e.g., ‘‘If I should find myself in a jam, I could think of many ways to get out of it’’ and ‘‘At the present time, I am energetically pursuing my goals’’). Response options ranged from 1 (definitely false) to 8 (definitely true). Higher average scale scores represent greater sense of agency. Coefficient alpha reliability was a = .91 | Based on multilevel models, youth who received more support/socializing, information/resources, and did more advocacy in their GSA reported greater agency. Support/socializing and advocacy distinctly contributed to agency even while accounting for the contribution of family support and positive LGBT school climate. Further, advocacy was associated with agency for sexual minority youth but not heterosexual youth. Greater organizational structure enhanced the association between support/socializing and agency; it also enhanced the association between advocacy and agency for sexual minority youth. These findings begin to provide empirical support for specific functions of GSAs that could promote wellbeing and suggest conditions under which their effects may be enhanced |
| Poteat VP, O’Brien MD, Rosenbach SB, Finch EK, Calzo JP. Depression, Anxiety, and Interest in Mental Health Resources in School-Based Gender-Sexuality Alliances: Implications for Sexual and Gender Minority Youth Health Promotion. Prevention Science. 2021;22(2):237-46. (USA) | Survey study with young people and advisors from GSAs in Massachusetts, on mental health generally, depression, and anxiety | Gay-Straight Alliances (GSAs) - what are the levels of depression and anxiety amongst GSA members, and how often is mental health discussed in these groups? | Participants were 580 youth (M age = 15.59 years, SD = 1.39 years; range = 10 to 20 years; 79% sexual minority; 57% cisgender female; 68% White) in 38 GSAs (4 to 34 students per GSA; M = 15 students, SD = 6.62) across Massachusetts and their advisors (n = 58; M age = 43.58 years, SD = 10.50 years; range = 27 to 62 years) | Depression (CESD-10 brief version); Anxiety (21-item Beck Anxiety Inventory) | Among youth, 70.1% scored above the threshold indicating probable mild depression, and 34.4% scored above the threshold suggesting concerning anxiety. Adjusted odds ratios indicated that the odds of depression and anxiety were higher for SGM members relative to heterosexual and cisgender members, particularly among youth reporting SGM identities that have been underrepresented. GSAs discussed mental health with some frequency over the school year. Youth and advisors expressed strong interest in resources. These findings support the case for developing selective and indicated school-based prevention programming for youth in GSAs to address their mental health needs |
| Poteat VP, Calzo JP, Yoshikawa H. Promoting youth agency through dimensions of gay–straight alliance involvement and conditions that maximize associations. Journal of Youth and Adolescence. 2016;45(7):1438-51. (Canada) | Secondary analyses of 2008 British Columbia Adolescent Health Survey | Gay-straight alliances and explicit anti-homophobic bullying policies | n =21,70 8. Most students identified themselves as 100% heterosexual (weighted n = 10,408 for boys; weighted n = 10,577 for girls) as compared to mostly heterosexual (weighted n = 840 for boys; weighted n = 914 for girls) or LGB (weighted n = 359 for boys; weighted n = 364 for girls) | Suicide-related variables—The suicide-related variables included serious suicidal ideation and the number of suicidal attempts in the past year. Despair—Despair, a proxy measure for depression symptoms, was assessed by asking if students had felt so sad, discouraged, hopeless, or had so many problems that they wondered if anything was worthwhile in the past 30 days | LGB students had lower odds of past year discrimination, suicidal thoughts, and attempts, mostly when policies and GSAs had been in place for 3+ years; policies had a less consistent effect than GSAs |
| Sandfort TG, Bos HM, Collier KL, Metselaar M. School environment and the mental health of sexual minority youths: A study among Dutch young adolescents. American journal of public health. 2010;100(9):1696-700. (Netherlands) | Survey questionnaire study: All schools are members of the VIOS (Veiligheid In en Om School, or Safety In and Around School) initiative, which explores ways to improve safety in and around the school environment for students in general | To explore whether participants’ ratings of support for cultural pluralism and consistency and clarity of rules at their schools were a reﬂection of actual differences between schools or were due to random variation, we conducted univariate analyses of covariance with schools as the independent factor, participants’ ratings of school climate as dependent variables, and gender, age, ethnicity, and social desirability as covariates to control for between-subject differences related to perceptions of school climate | *n=*513 adolescents (12 to 15 years old; Mean - 14.02 years); 43.3% male; 55.6% Dutch ethnic background; 99% reported having experienced same-sex attractions | Mental health was assessed with items from a shortened version of the Brief Symptom Inventory, asking about the occurrence of 24 symptoms in the preceding week | Adolescents with same-sex attractions in schools where rules and expectations were experienced as less consistent and clear reported signiﬁcantly more mental health problems than their peers with no same-sex attractions in the same schools. Such differences were absent in schools where rules and expectations were experienced as more consistent and clear. There were no such effects of cultural pluralism. Results suggest that schools with consistent and clear rules and expectations mitigate the risk for mental health problems among students with same-sex attractions and underscore the importance of structural measures for the health of sexual minority youth |
| Zhang L, Finan LJ, Bersamin M, Fisher DA. Sexual orientation–based depression and suicidality health disparities: The protective role of school‐based health centres. Journal of research on adolescence. 2020;30:134-42. (USA) | Secondary analyses of Oregon Healthy Teens Survey and School Sample | School-based health centres; examines whether sexual orientation-based differences in mental health indicators were smaller in schools with SBHCs than those without SBHCs among a representative sample of Oregon adolescents | The current study is based on 13,608 students in 137 public high schools in Oregon that participated in the OHT Survey in 2015. A total of 26 of these schools had SBHCs at this time. The population of Oregon is predominately White (87%) and non-Hispanic (75%), with only 13% of the population living in poverty | Students were asked three questions about their feelings, thoughts, and behaviours related to depressed mood and suicide | Regression results revealed significant SBHC by SMY status interactions indicating relative reductions in likelihood of depressive episodes (30%), suicidal ideation (34%), and suicide attempts (43%) among SMY in schools with SBHCs. SMY students in SBHC schools reported lower likelihood of a past-year depressive episode, suicidal ideation, and suicide attempt versus those attending non-SBHC schools. Conversely, no differences in these outcomes were observed for non-SMY by SBHC status |
